# Identification of Hot Spots in Protein Structures Using Gaussian Network Model and Gaussian Naive Bayes

**DOI:** 10.1155/2016/4354901

**Published:** 2016-11-02

**Authors:** Hua Zhang, Tao Jiang, Guogen Shan

**Affiliations:** ^1^School of Computer and Information Engineering, Zhejiang Gongshang University, Hangzhou, Zhejiang 310018, China; ^2^School of Statistics and Mathematics, Zhejiang Gongshang University, Hangzhou, Zhejiang 310018, China; ^3^School of Community Health Sciences, University of Nevada Las Vegas, Las Vegas, NV 89154, USA

## Abstract

Residue fluctuations in protein structures have been shown to be highly associated with various protein functions. Gaussian network model (GNM), a simple representative coarse-grained model, was widely adopted to reveal function-related protein dynamics. We directly utilized the high frequency modes generated by GNM and further performed Gaussian Naive Bayes (GNB) to identify hot spot residues. Two coding schemes about the feature vectors were implemented with varying distance cutoffs for GNM and sliding window sizes for GNB based on tenfold cross validations: one by using only a single high mode and the other by combining multiple modes with the highest frequency. Our proposed methods outperformed the previous work that did not directly utilize the high frequency modes generated by GNM, with regard to overall performance evaluated using *F*1 measure. Moreover, we found that inclusion of more high frequency modes for a GNB classifier can significantly improve the sensitivity. The present study provided additional valuable insights into the relation between the hot spots and the residue fluctuations.

## 1. Introduction

Flexibility and dynamics play key roles for proteins in implementing various biological processes and functions [1, 2]. Residue fluctuations or atomic motions, contributing to large-scale conformational changes of protein structures, are shown to be closely related to functions of native proteins [3–5].

Two methods, molecular dynamic (MD) simulation and normal mode analysis (NMA), are widely used to investigate the dynamic link between protein structures and functions. The main drawback of MD simulations is their computational cost [6, 7]. Coarse-grained NMA, such as elastic network model (ENM) [7], has been increasingly used in recent years as a powerful tool to elucidate the structure-encoded dynamics of biomolecules [2]. The ENMs, including the isotropic Gaussian network model (GNM) [8, 9] and the anisotropic network model [10], define spring-like interactions between residues that are within a certain cutoff distance. They simplify the computationally costly all-atom potentials into a quadratic function in the vicinity of the native state, which allows the decomposition of the motions into vibrational modes with different frequencies that are often known as normal modes. Being simple and efficient, ENM and GNM have been validated in numerous applications that resulted in reasonable agreement with a wealth of experimental data, including prediction of X-ray crystallographic B-factors for amino acids [9, 11], identifications of hot spots [12–14], catalytic sites [15], core amino acids stabilizing rhodopsin [16] and important residues of HLA proteins [17], elucidation of the molecular mechanisms of motor-protein motions [18], and general conformational changes and functions [3, 4, 19–31].

Previous studies have shown in many cases that the normal modes including the high frequency (fast) modes and the low frequency (slow) modes by the GNM are very useful for recognizing several specific types of protein functions. In particular, the highest frequency modes that reflect local events at the residue level can be utilized to identify core residues or binding sites [16, 17, 20, 32], while the lowest frequency modes are usually responsible for the collective functional dynamics of the global protein motions [23, 33]. In area of protein-protein interaction, several studies such as Ozbek et al. [12], Haliloglu et al. [13], and Demirel et al. [14] utilized GNM to identify hot spots that are defined as the residues contributing more than 2 kcal/mol to the binding energy. Their results suggested that hot spots are predefined in the dynamics of protein structures and forming the binding core of interfaces. However, the mean square distance fluctuations of residue pairs and the mean square fluctuations of residues calculated from the highest frequency modes by GNM, rather than the direct usage of the highest frequency modes themselves, were applied to detect the hot spots in the work by Ozbek et al. [12] and by Haliloglu et al. [13] and Demirel et al. [14], respectively.

In addition, several computational methods by utilizing machine learning tools have been developed to predict hot spots from protein sequences and structures [34–37]. The advantage of learning methods is the ability to result in higher quality by sufficiently integrating the extracted feature information from protein structures. In this paper, we follow the work by Ozbek et al. [12] but focus on the direct usage of the highest frequency modes to investigate the relation between the residue fluctuations and the hot spots. The top 20 highest frequency modes by GNM were used as an original feature set inputted into Gaussian Naive Bayes (GNB), as a representative of learning methods, to identify hot spots. The main purpose of this study is to examine whether the raw fast modes can be directly used to differentiate hot spots or non-hot spots and whether the utilization of learning methods can improve the identification quality of hot spots for unbound protein structures.

## 2. Material and Methods

### 2.1. Dataset

We used the dataset that was collected by Ozbek et al. [12]. This set was filtered with PISCES culling server [38] at the sequence identity of 25% and was originally composed of 33 unbound protein structures. We had to remove one protein with ID 1lrp from the dataset since its structure cannot be currently found in Protein Data Bank (PDB) [39]. Therefore, the final dataset had 32 unbound protein structures with a total of 4270 residues of which 171 are hot spot residues. The dataset including the detailed information about hot spot residues can be derived from Ozbek et al. [12].

### 2.2. Gaussian Network Model and Its Applications to Identification of the Hot Spots

GNM describes each protein as an elastic network, where the springs connecting the nodes represent the bonded and nonbonded interactions between the pairs of residues located within a cutoff distance *R*_*C*_ [8, 9]. Assuming that the springs are harmonic and the residue fluctuations are isotropic and Gaussian, the network potential of *N* nodes (residues) in a protein structure is

(1) $$\begin{array}{c} V_{GNM}=\frac{\gamma }{2}\overset{N}{\underset{i,j}{\sum }}\Gamma_{ij}{\left(\;R_{ij}-R_{ij}^{0}\;\right)}^{2}, \end{array}$$

where **R**_*ij*_ and **R**_*ij*_^0^ are instantaneous and original distance vectors between residues *i* and *j*, respectively, *γ* is the force constant assumed to be uniform for all network springs, and Γ = (Γ_*ij*_) is the Kirchhoff connectivity matrix defined as

(2) $$\begin{array}{c} \Gamma_{ij}=\left\{\begin{array}{cc} -1, & \text{if }i\neq j\text{ and }\;R_{ij}^{0}\leq R_{C}\\ 0, & \text{if }i\neq j\text{ and }R_{ij}^{0}\geq R_{C}\\ -\sum_{j:j\neq i}\Gamma_{ij}, & \text{if }i=j, \end{array}\right. \end{array}$$

where *R*_*ij*_^0^ is the distance between residues *i* and *j* and *R*_*C*_ is given as a cutoff. Then, the mean correlation between residue fluctuations is calculated as

(3) ΔRi·ΔRj3kBTγΓ−1ij=3kBTγUΛ−1UTij,

where** U** is the orthogonal matrix of eigenvectors (**u**_*i*_), Λ is the diagonal matrix of eigenvalues (*λ*_*i*_), *k*_*B*_ is the Boltzmann constant, and *T* is the absolute temperature.

To identify hot spot residues, Ozbek et al. [12] used the mean square distance fluctuations (MSDF), 〈Δ**R**_*ij*_^2^〉, of residues *i* and *j* given as

(4) ΔRij2ΔRi−ΔRj2=ΔRi2+ΔRj2−2ΔRi·ΔRj,

which were calculated using high frequency modes of GNM based on a cutoff of 6.5 Å. The residues with relatively high MSDF value were considered functionally probable; see more details in Ozbek et al. [12].

In addition, both Haliloglu et al. [13] and Demirel et al. [14] similarly defined mean square fluctuation (or vibration) (MSF) of residues in the weighted average of several high frequency modes based on a cutoff of 7.0 Å, to identify the hot spot residues. The MSF of residue *i* weighed by a subset of modes *k*_1_ ≤ *k* ≤ *k*_2_ is given as

(5) $$\begin{array}{c} {\langle \;\Delta R_{i}^{2}\;\rangle }_{k_{1}-k_{2}}=\frac{\left(3k_{B}T/\gamma \right)\sum_{k=k_{1}}^{k_{2}}\lambda_{k}^{-1}{\left[u_{k}\right]}_{i}^{2}}{\sum_{k=k_{1}}^{k_{2}}\lambda_{k}^{-1}}. \end{array}$$

Then, one residue was predicted as a hot spot if the normalized MSF of the residue (i.e., the measure expressed in (5) divided by 3*k*_*B*_ *T*/*γ*) is larger than a given threshold. The main difference between the work by Haliloglu et al. [13] and that by Demirel et al. [14] is the different thresholds adopted. Haliloglu et al. [13] used a constant threshold of 0.005 while it was 6*N* ^−1^ given by Demirel et al. [14] where *N* is the number of residues in a protein sequence.

### 2.3. Gaussian Naive Bayes

A Naive Bayes (NB) classifier calculates the probability of a given instance (example) belonging to a certain class [40]. Given an instance *X* described by its feature vector (*x*_1_,…, *x*_*n*_) and a class target *y*, the conditional probability *P*(*y*∣*X*) can be expressed as a product of simpler probabilities using the Naive independence assumption according to Bayes' theorem:

(6) $$\begin{array}{c} P\left(\;y\;|\;X\;\right)=\frac{P\left(y\right)P\left(X\;|\;y\right)}{P\left(X\right)}=\frac{P\left(y\right)\prod_{i=1}^{n}P\left(x_{i}\;|\;y\right)}{P\left(X\right)}. \end{array}$$

Here, the target *y* may have two values where *y* = 1 means a hot spot residue and *y* = 0 represents non-hot spot residue. *X* for one residue (one instance) is a feature vector with the same size for describing its characteristic using high frequency modes generated by GNM. For example, *X* is equal to a vector composed of *i*th component **u**_*ki*_ for *i*th residue in a sequence when only one high frequency mode **u**_*k*_ is used. If three high frequency modes, denoted by **u**_1_, **u**_2_, and **u**_3_, are taken into account, the vector *X* will be (**u**_1*i*_, **u**_2*i*_, **u**_3*i*_) for residue *i* in a protein sequence. Moreover, if a window size of 3 with respect to the residue *i* is adopted, *X* becomes (**u**_1*i*−1_, **u**_1*i*_, **u**_1*i*+1_, **u**_2*i*−1_, **u**_2*i*_, **u**_2*i*+1_, **u**_3*i*−1_, **u**_3*i*_, **u**_3*i*+1_).

Since *P*(*X*) is constant for a given instance, the following rule is adopted to classify the instance whose class is unknown:

(7) $$\begin{array}{c} \hat{y}=\arg\underset{y}{\max}\;P\left(\;y\;\right)\overset{n}{\underset{i=1}{\prod }}P\left(\;x_{i}\;|\;y\;\right), \end{array}$$

where “arg” means a value of *y* so that the above expression is maximized; that is, if *P*(*y* = 1)∏_*i*_ *P*(*x*_*i*_∣*y* = 1) is larger than *P*(*y* = 0)∏_*i*_ *P*(*x*_*i*_∣*y* = 0), $\hat{y}=1$; otherwise, $\hat{y}=0$.

Moreover, when the likelihood of the features (i.e., *P*(*x*_*i*_∣*y*)) is assumed to be Gaussian, a NB classifier is called Gaussian Naive Bayes (GNB). Due to its simplicity and being computationally fast compared to other more sophisticated methods, GNB has been widely applied to prediction problems in bioinformatics [41, 42]. In this study, GNB was mainly used to train the models by inputting the highest frequency modes to identify hot spot residues.

### 2.4. Performance Evaluation

In a classification task, the following quality indices, including sensitivity (also known as recall), specificity, precision, and the overall accuracy, were generally used to assess prediction performance:

(8) Sensitivity:  sen=TPTP+FN,Specificity:  spe=TNTN+FP,Precision:  pre=TPTP+FP,Accuracy:  acc=TP+TNTP+TN+FP+FN,

where true positives (TP) and true negatives (TN) correspond to correctly predicted hot spot residues and non-hot spot residues, respectively, false positives (FP) denote non-hot spot residues predicted as hot spot residues, and false negatives (FN) denote hot spot residues predicted as non-hot spot residues.

Obviously, the dataset used in this study is extremely unbalanced with a very high proportion of non-hot spot residues. For this reason, the accuracy value is not a good choice to evaluate the overall performance of results. When a dataset includes 95% negative samples but 5% positive samples, a classifier may identify all of them as negative, resulting in 95% overall accuracy and 100% specificity. This is really shown as excellent performance, but it fails to identify the positive samples that we actually need pay close attention to. Moreover, two indices, sensitivity and precision, can both measure the classification correctness for positive samples. It is strongly expected that these two indices can synchronously reach high values, but there exists a trade-off between them in general. Therefore, we used *F*1 measure to evaluate the overall prediction performance:

(9) $$\begin{array}{c} F1\text{ measure: }F1=\frac{2\times sen\times pre}{sen+pre}, \end{array}$$

which can balance the sensitivity and the precision in case of the unbalanced dataset. The formula of the *F*1 measure can be changed to be *F*1 = 2/((1/sen)+(1/pre)) when both sen and pre are exactly larger than zero. Thus, *F*1 measure can be viewed as an increasing function of sen and pre. The minimum of *F*1 is 0 when sen = 0 or pre = 0, and the maximum of *F*1 is 1 when sen = 1 and pre = 1.

### 2.5. Identification of Hot Spots Using GNM and GNB

The experimental performance on identification of hot spot residues is tested using *n*-fold cross validation (*n*CV) on the dataset composed of 32 unbound protein structures. In the *n*CV procedure, chains are randomly divided into *n* subsets with the same numbers of sequences, and the test is repeated *n* times. In each time, the *n* − 1 subsets are used to build the model, and the remaining one subset is then tested by the prediction model.

In the present study, we performed tenfold cross validation (10CV) based on Gaussian Naive Bayes using the highest modes as features from GNM outputs in different ways. Then, we mainly implemented two schemes concerning feature coding for investigating the relations between the highest modes and the hot spot residues. Firstly, a classifier was modeled by directly using single one of the top 20 high frequency modes (i.e., the eigenvectors (**u**_*i*_) that correspond to the top 20 largest eigenvalues (*λ*_*i*_)). Meanwhile, a sliding window of the central residue with sizes ranging from 1 to 21 was utilized to examine the impact of the neighboring residues' fluctuations, and the computation of GNM was carried out by usage of multiple distance cutoffs ranging from 6.0 to 8.0 with a step size of 0.1. Secondly, we combined top *m* modes with the highest frequency (*m* = 1,2, 3,…, 20) and utilized similar scheme for the distance cutoff of GNM computation and the sliding window of the central residue to establish the models for identifying hot spot residues.

## 3. Results and Discussion

### 3.1. Identification of Hot Spot Residues Using Single One of the Highest Modes

In this work, the overall performance was evaluated by the *F*1 measure in (9), which is able to balance the sensitivity and the precision.  Table 1 lists twenty computational outcomes of the prediction performance that are ordered by *F*1 measure, where the feature vector for a GNB classifier was extracted from single one mode, that is, *i*th highest mode (*i* = 1,2,…, 20), the distance cutoff in GNM varied from 6.0 to 8.0 with the step size of 0.1, and the sliding window for one mode ranged from 1 to 21 with a step size of 2. As shown in Table 1, the highest performance was achieved by *F*1 measure of 0.1517 when the distance cutoff is 7.1 Å and the size of the sliding window is 3 in case of the 8th highest mode.

Moreover, top six *F*1 measures shown in Table 1 were from the same 8th highest mode, indicating that the best performance achieved may not belong to the first or second highest frequency mode. Even the 19th and the 13th highest modes can also result in relatively high *F*1 measures. From the aspect of cutoff, it has been shown that majority of the cutoff values shown in Table 1 are in or close to the [7.0, 7.3] interval.

**Figure 1:**
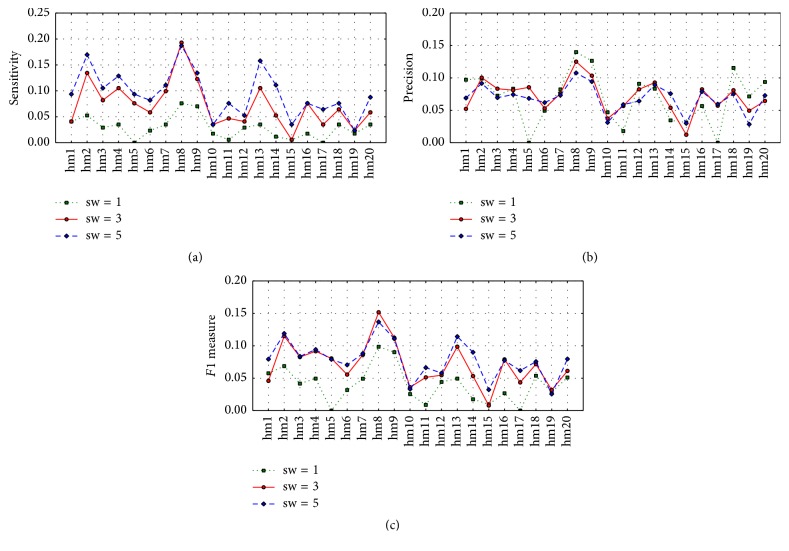
Plots of sensitivity (a), precision (b), and *F*1 values by the single *i*th highest mode (*i* = 1,2,…, 20) in three cases of the sliding window sizes (sw) (i.e., sw = 1, 3, 5) for GNB classifiers. The *i*th highest mode in the figure is denoted as hm*i*.

Given the cutoff of 7.3 Å in GNM, we plotted sensitivity, precision, and *F*1 measure for all of the top 20 high modes; see Figure 1. Three cases with sizes of the sliding windows equal to 1, 3, and 5 were examined. It is apparent that the *F*1 measures and the sensitivity values for the majority of the 20 modes can be improved when the size of the sliding window is from 1 to 3. However, there is no sufficient evidence to prove that larger size of the sliding window can further increase the *F*1 measure. On the other hand, the majority of the sensitivity values were improved when the window size was increased from 3 to 5, but no consistent trend can be found for precision values in three cases of the window sizes.

### 3.2. Identification of Hot Spot Residues by Combining the Highest Modes

Furthermore, top *m* modes (*m* = 1,2,…, 20) with the highest frequency were combined to establish the GNB classifier and to investigate whether the prediction performance can be improved. For example, when *m* is taken to be 10, top ten high modes (i.e., hm1, hm2,…, hm10) are together inputted into the feature vector of a GNB classifier. Meanwhile, the classification experiments were also performed on various cases in which the distance cutoff is from 6.0 to 8.0 with the step size of 0.1 and the size of the sliding window (sw) ranges from 1 to 21 with the step size of 2.  Table 2 lists twenty outcomes of these computational experiments ordered by *F*1 measure. Among these results, the size of the sliding window is almost 1 except the case of the 10th highest *F*1 measure in which 9 high modes and the window size of 3 were used, suggesting that the fluctuation of the central residue may be sufficient to identify hot spot residues by a combination of multiple high frequency modes. Moreover, as shown in Table 2, the distance cutoff often belongs to the [7.1, 7.5] interval, and it seems that a larger *m* value tends to result in higher sensitivity. For instance, the sensitivity value obtained by a combination of the top 10 high modes with cutoff of 7.4 Å (i.e., the case of top 1 *F*1 measure) is 0.2924, while the sensitivity values in the cases of top 4, 6, and 7 *F*1 measures, which are achieved by the usage of the top 20, 19, and 20 high modes, respectively, are all larger than 0.41.

**Figure 2:**
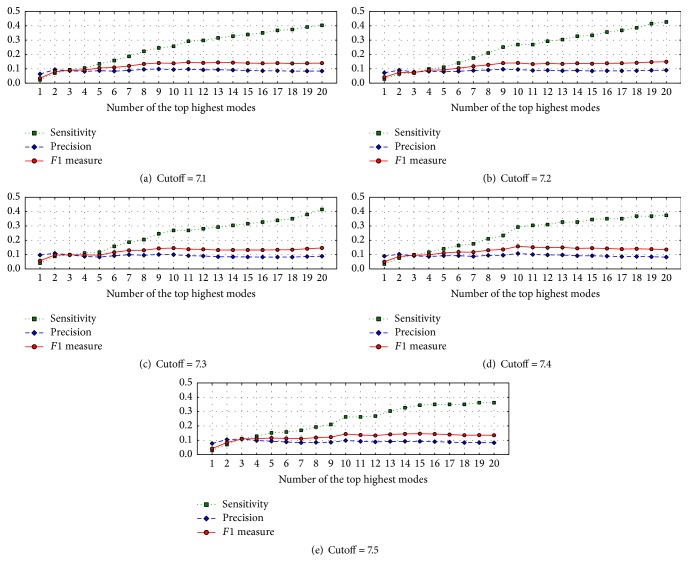
Plots of sensitivity, precision, and *F*1 values against *m* modes with the highest frequency in five cases denoted by the distance cutoffs of 7.1 Å (a), 7.2 Å (b), 7.3 Å (c), 7.4 Å (d), and 7.5 Å (e), respectively, for GNB classifiers.

In Figure 2, we plotted the sensitivity, the precision, and the *F*1 measure against *m* modes with the highest frequency that were combined as features for five cases denoted by the distance cutoffs of 7.1 Å, 7.2 Å, 7.3 Å, 7.4 Å, and 7.5 Å, respectively, where the sizes of the sliding window for all cases are 1. It can be seen from the figure that these three indices are consistently improved with the number of top high modes used up to 10. Especially for the case of sensitivity, its value is an increasing function of the number of modes with the highest frequency. It can be concluded that inclusion of more high frequency modes can improve the sensitivity, but the precision values become slightly decreased by adding more high frequency modes when the number of high modes combined is larger than 10. In the meantime, the *F*1 measure tends to be no longer enhanced.

### 3.3. Performance Comparison with Existing Methods

In the present work, we directly inputted the high frequency modes to a GNB classifier for predicting hot spots when compared with the existing methods proposed by Ozbek et al. [12], Haliloglu et al. [13], and Demirel et al. [14]. Ozbek et al. [12] utilized the mean square distance fluctuations of residue pairs, which were computed at most based on five top high frequency modes, to identify hot spot residues. It may be not appropriate to directly compare our work with the results obtained by Ozbek et al. [12], since the datasets used and the test procedures are both slightly different. However, we reported here again part of outcomes from Table 1 in Ozbek et al. [12] for a comparison. The *F*1 measures were calculated on the reported sensitivity and precision values, as shown in Table 3. In addition, no results concerning the prediction quality of hot spot residues based on a nonredundant dataset were reported in Haliloglu et al. [13] and Demirel et al. [14], where only MSF profiles for a couple of protein cases were depicted and shown as figures. The usage of the number of high frequency modes is not consistent that three, four, or five fast modes may be adopted for different cases. Due to the lack of details and web servers, we here simulated their methods on the dataset in this work by computing the normalized MSF values weighted by one up to five high frequency modes using a cutoff of 7 Å for GNM. A constant 0.005 and a varied value 6*N* ^−1^ with respect to the sequence length *N* were used to identify hot spot residues for the simulations of the methods by Haliloglu et al. [13] and Demirel et al. [14], respectively. The quality indices including sensitivity, specificity, precision, accuracy, and *F*1 measure for these simulations were then reported in Table 3. We also listed part of the best outcomes from this study in Table 3, two using single high mode and four by a combination of multiple high modes, which have been shown in Tables 1 and 2.

On the whole, if evaluated by *F*1 measure or precision, all of the cases in Table 3 by this work outperformed the results by Ozbek et al. [12] and by the simulated methods of Haliloglu et al. [13] and Demirel et al. [14]. This suggests that the direct usage of the high frequency modes is efficient to identify hot spot residues. Besides, the improvement on *F*1 measure by combining multiple high frequency modes seems to be very slight when compared with the methods only using single high mode, while the sensitivity values in general tend to be improved a lot. This is in good agreement with the work by Ozbek et al. [12] and the simulation results of Haliloglu et al. [13] and Demirel et al. [14] as outlined in Table 3. Additionally, the specificity and accuracy values of the simulated method for Demirel et al. [14] are higher than those of other methods, but on the contrary the values of sensitivity, precision, and *F*1 measure are in general lower. The reason causing worse quality on *F*1 measure achieved by the simulation of Demirel et al. [14] is due to a larger threshold used when compared with the simulated method of Haliloglu et al. [13].

In addition, we also performed computational experiments using several common classifiers, including logistic regression, decision tree, *k*-nearest neighbor, and support vector machine with default parameters, instead of GNB, where all of the machine learning methods were implemented in scikit-learn [43]. As a consequence, the results (data not shown in this paper) showed that GNB exhibited better performance than other classifiers. This is the reason why we finally adopted GNB as the base classifier for identification of the hot spot residues.

## 4. Conclusion

In this study, we followed previous work [12–14] focusing on the identifications of hot spots by using GNM but directly used the high frequency modes and further performed GNB classifier. The proposed methods outperformed the outcomes reported in Ozbek et al. [12] and the simulated results of Haliloglu et al. [13] and Demirel et al. [14] based on *F*1 measure to evaluate the overall performance. The results by this work suggested that the high frequency modes can be directly used to identify hot spot residues with reasonable performance. In case of the scheme using only single high frequency mode, the largest *F*1 measure may not be necessarily achieved by one of the top five high frequency modes. In our study, it was surprisingly gained by the 8th highest mode with the distance cutoff of 7.3 and the window size of 3. We further included more modes from total number of 20 high frequency modes when compared with the work by Ozbek et al. [12] in which at most five frequency modes are used. Of particular interest is the fact that inclusion of more high frequency modes can significantly improve the sensitivity value, but not the *F*1 measure and the precision in general.

The dataset used in this work is obviously unbalanced. There is a trade-off between the sensitivity and the precision. It is not easy for researchers to find a perfect way to determine the proper performance index to evaluate experimental results. Therefore, we finally reported multiple results as listed in Tables 1, 2, and 3, which were considered for choices associated with different purposes in practice. Overall, the present study provided additional valuable insight into the relation between hot spots and residue fluctuations.

## Figures and Tables

**Table 1:** List of top 20 *F*1 measures based on tenfold cross validations of Gaussian Naive Bayes when using single *i*th highest mode (*i* = 1,2,…, 20) inputted into the feature vector, where cutoff means the distance threshold for GNM computation that varies from 6.0 to 8.0 with step size of 0.1 and sw represents the size of the sliding window for the central residue that ranges from 1 to 21 with step size of 2.

Top	Cutoff	*i*	sw	sen	spe	pre	acc	*F*1 measure
1	7.3	8	3	0.1930	0.9436	0.1250	0.9136	**0.1517**
2	7.1	8	9	0.2515	0.9095	0.1039	0.8831	0.1470
3	7.1	8	7	0.2456	0.9119	0.1042	0.8852	0.1463
4	7.1	8	5	0.2164	0.9263	0.1091	0.8979	0.1451
5	7.1	8	3	0.1696	0.9473	0.1184	0.9162	0.1394
6	7.3	8	5	0.1871	0.9354	0.1077	0.9054	0.1368
7	8.0	3	5	0.1930	0.9310	0.1044	0.9014	0.1355
8	7.3	8	7	0.2164	0.9163	0.0974	0.8883	0.1343
9	7.1	8	13	0.2281	0.9090	0.0947	0.8817	0.1338
10	7.1	8	11	0.2281	0.9071	0.0929	0.8799	0.1320
11	7.0	19	17	0.2456	0.8963	0.0899	0.8703	0.1317
12	6.7	13	3	0.1345	0.9619	0.1285	0.9288	0.1314
13	7.8	3	3	0.1520	0.9507	0.1140	0.9187	0.1303
14	7.0	14	21	0.2339	0.901	0.0897	0.8742	0.1297
15	7.0	19	19	0.2456	0.8934	0.0877	0.8674	0.1292
16	7.1	8	15	0.2281	0.9039	0.0901	0.8768	0.1291
17	7.0	4	7	0.2281	0.9022	0.0886	0.8752	0.1277
18	6.6	6	3	0.1520	0.9480	0.1088	0.9162	0.1268
19	6.9	15	21	0.2222	0.9046	0.0886	0.8773	0.1267
20	7.2	14	13	0.2456	0.8897	0.0850	0.8639	0.1263

**Table 2:** List of the top 20 *F*1 measures based on tenfold cross validations of Gaussian Naive Bayes when using *m* modes with the highest frequency inputted into the feature vector, where *m* = {1,2,…, 20}, the distance cutoff in GNM varies from 6.0 to 8.0 with step size of 0.1, and the sliding window size (sw) for multiple high modes ranges from 1 to 21 with step size of 2.

Top	Cutoff	*m*	sw	sen	spe	pre	acc	*F*1 measure
1	7.4	10	1	0.2924	0.8992	0.1080	0.8749	**0.1577**
2	7.4	11	1	0.3041	0.8873	0.1012	0.8639	0.1518
3	7.4	13	1	0.3275	0.8736	0.0976	0.8518	0.1503
4	7.2	20	1	0.4269	0.8207	0.0903	0.8049	0.1491
5	7.4	12	1	0.3099	0.8809	0.0980	0.8581	0.1489
6	7.2	19	1	0.4152	0.8239	0.0895	0.8075	0.1473
7	7.3	20	1	0.4152	0.8229	0.0891	0.8066	0.1467
8	7.1	11	1	0.2924	0.8870	0.0975	0.8632	0.1462
9	7.5	15	1	0.3450	0.8592	0.0928	0.8386	0.1462
10	7.1	9	3	0.3977	0.8312	0.0895	0.8138	0.1461
11	7.3	10	1	0.2690	0.8992	0.1002	0.8740	0.1460
12	7.4	15	1	0.3450	0.8585	0.0923	0.8379	0.1457
13	7.1	13	1	0.3158	0.8727	0.0937	0.8504	0.1446
14	7.5	14	1	0.3275	0.8663	0.0927	0.8447	0.1445
15	7.5	16	1	0.3509	0.8529	0.0905	0.8328	0.1439
16	7.4	14	1	0.3275	0.8653	0.0921	0.8438	0.1438
17	7.6	15	1	0.3333	0.8622	0.0916	0.8410	0.1438
18	7.5	10	1	0.2632	0.9000	0.0989	0.8745	0.1438
19	7.3	9	1	0.2456	0.9090	0.1012	0.8824	0.1433
20	7.1	14	1	0.3275	0.8641	0.0914	0.8426	0.1429

**Table 3:** Performance comparison of the proposed models with the work by Ozbek et al. [12] and the simulated methods proposed by Haliloglu et al. [13] and Demirel et al. [14], where hm1–*i* means that a total of *i* high frequency modes (hm1, hm2,…, hm*i*) are used together.

Reference	GNM modes	Cutoff	sw	sen	spe	pre	acc	*F*1
Ozbek et al. [12]	hm1			0.14	0.89	0.05	0.86	0.0737
	hm2			0.16	0.80	0.05	0.85	0.0762
	hm3	6.5 Å	1	0.24	0.88	0.07	0.85	0.1084
	hm1–3			0.25	0.86	0.07	0.83	0.1094
	hm1–5			0.29	0.84	0.07	0.81	0.1128

Haliloglu et al. [13] (simulated)	hm1			0.1988	0.9019	0.0780	0.8738	0.1120
	hm1-2			0.2690	0.8819	0.0868	0.8574	0.1312
	hm1–3	7.0 Å	1	0.3041	0.8580	0.0820	0.8358	0.1292
	hm1–4			0.3275	0.8429	0.0800	0.8222	0.1286
	hm1–5			0.3450	0.8339	0.0797	0.8143	0.1295

Demirel et al. [14] (simulated)	hm1			0.0468	**0.9773**	0.0792	**0.9400**	0.0588
	hm1-2			0.0526	0.9697	0.0677	0.9330	0.0592
	hm1–3	7.0 Å	1	0.0409	0.9615	0.0424	0.9246	0.0417
	hm1–4			0.0819	0.9573	0.0741	0.9222	0.0778
	hm1–5			0.0936	0.9532	0.0769	0.9187	0.0844

This work	hm8	7.3 Å	3	0.1930	0.9436	**0.1250**	0.9136	0.1517
	hm8	7.1 Å	9	0.2515	0.9095	0.1039	0.8831	0.1470
	hm1–10	7.4 Å	1	0.2924	0.8992	0.1080	0.8749	**0.1577**
	hm1–11	7.4 Å	1	0.3041	0.8873	0.1012	0.8639	0.1518
	hm1–13	7.4 Å	1	0.3275	0.8736	0.0976	0.8518	0.1503
	hm1–20	7.2 Å	1	**0.4269**	0.8207	0.0903	0.8049	0.1491
